# The diversity of viral community in *Sogatella furcifera* revealed by meta-transcriptomics

**DOI:** 10.3389/fmicb.2025.1617239

**Published:** 2025-06-18

**Authors:** Jihan Wang, Yu Zhu, Dongyuan Li, Xinyue Zheng, Chunlian Chai, Jie Zhang, Jianguo Wu, Qun Hu, Shanshan Zhao

**Affiliations:** ^1^State Key Laboratory of Agriculture and Forestry Biosecurity, Center for Genetic Improvement, Vector-borne Virus Research Center, College of Plant Protection, Fujian Agriculture and Forestry University, Fuzhou, China; ^2^Yunnan Provincial Key Lab of Agricultural Biotechnology, Institute of Biotechnology and Germplasm Resources, Yunnan Academy of Agricultural Sciences, Kunming, China

**Keywords:** RNA viromes, *Sogatella furcifera*, *Sobelivirales*, vsiRNA, SoSNV1

## Abstract

**Introduction:**

Metagenomic analyses has significantly advanced our understanding of viral evolution and their functions within organismal biology. In particular, exploring the virome of agricultural pests like the white-backed planthopper (WBPH) is essential for understanding their role as potential virus vectors and developing effective pest management strategies.

**Methods:**

To explore viral diversity, we collected white-backed planthoppers (WBPHs) from nine sites spanning four Chinese provinces (Liaoning, Fujian, Guangxi, and Yunnan) and performed metagenomic sequencing.

**Results:**

Our analysis identified 11 novel viruses belonging to 7 viral families, encompassing positive-sense single-stranded RNA (+ssRNA), negative-sense single-stranded RNA (-ssRNA), and double-stranded RNA (dsRNA) viruses. Remarkably, eight of the southern Chinese sites, excluding one in Liaoning province, contained a previously undiscovered *Sobelivirales* virus. Using rapid-amplification of cDNA ends (RACE), we determined the complete genome sequence of this novel *Sobelivirales* virus. Subsequent analyses of its encoded proteins, potential structural domains, and phylogenetic relationships suggested that it may belong to a new genus within the *Sobelivirales*. Small RNA sequencing confirmed viral replication in WBPH by revealing that virus-derived small interfering RNAs (vsiRNAs) were primarily 21 and 22 nucleotides long.

**Discussion:**

Our results have important implications for understanding virus carriage in WBPHs, evaluating their role as virus vectors, and informing the development of improved pest management strategies. Furthermore, this study highlights the power of metagenomics in uncovering novel viruses and expanding our knowledge of viral diversity.

## Introduction

Viruses are obligate intracellular pathogens that infect all living cells. Effective containment of viral outbreaks necessitates both robust preparedness and swift response to emerging and recurring infections. Accurate identification of the causative agent is a pivotal step in effectively addressing disease outbreaks (Correa et al., 2021; French and Holmes, 2020). While virologists have historically focused on viruses affecting humans, domestic animals, and crops, recent advancements in metagenomics, particularly high-throughput sequencing of ecological samples, have unveiled a vast and diverse virome distributed across the biosphere (Cobbin et al., 2021; Hasiow-Jaroszewska et al., 2021; Simmonds et al., 2017; Wolf et al., 2020).

Insect-borne viruses are prevalent in both managed and natural ecosystems, posing significant economic threats to agriculture and forestry through their role as agents of destructive diseases (Eigenbrode et al., 2018; Marques and Imler, 2016; Ohlund et al., 2019; Patterson et al., 2020). Crop viral diseases transmitted by insect-borne viruses are major contributors to global reductions in crop yield and quality (He and Creasey Krainer, 2020; Jones, 2021; Wu et al., 2022). These diseases not only compromise food security through significant reductions in crop yields, but also inflict substantial economic losses (Nicaise, 2014; Savary et al., 2019). The absence of a systematic and comprehensive understanding of pathogen virulence and plant resistance mechanisms has hindered effective monitoring of vector insect migration and disease outbreaks (Heck and Brault, 2018; Mehetre et al., 2021; Wu et al., 2024). Furthermore, the origins and evolutionary trajectories of plant pathogens remain largely unknown (Lefeuvre et al., 2019; Rubio et al., 2020). Consequently, the development of comprehensive strategies for the effective control of plant viral diseases remains elusive.

Rice, a staple food crop in China, is subjected to various biotic and abiotic stresses during its growth cycle, with viral diseases posing significant threats (Zhao et al., 2021). All economically significant rice viruses are insect-borne, especially those transmitted by planthopper insects, including brown planthopper (BPH, *Nilaparvata lugens*), white-backed planthopper (WBPH, *Sogata furcifera*), and small brown planthopper (SBPH, *Laodelphax striatella*) (Otuka, 2013; Uehara-Ichiki et al., 2013; Wang et al., 2022; Wei and Li, 2016). Among them, the WBPH is a globally distributed insect, prevalent in regions such as Central, Eastern, and Southern Asia, as well as Northern Oceania. The WBPHs primarily affects Gramineae plants and is a vector for multiple viruses, notably the Southern rice black-streaked dwarf virus (SRBSDV), which currently poses a significant threat to rice production (Zhou et al., 2013). Current research predominantly focuses on the investigation and early detection of known viruses, with few studies addressing the prediction of new virus outbreaks within the WBPH population.

To characterize the virome of white-backed planthoppers, we performed metavirome sequencing on samples collected from nine sites spanning four Chinese provinces: Liaoning, Yunnan, Fujian, and Guangxi. Our analysis of the abundant viral sequences revealed 28 nearly complete viral genomes, of which 11 were novel. A novel *Sobelivirales* virus was identified in WBPH samples from eight of the nine locations studied, excluding Liaoning province. This virus has been tentatively named *Sogatella furcifera* solemo-like virus 1 (SoSNV1). Using rapid-amplification of cDNA ends (RACE), we determined the full-length genome of the virus to be 3,145 nucleotides, encoding at least four proteins. Phylogenetic analysis suggests that the virus is closely related to sobemo-like virus, indicating its potential as a novel plant pathogen transmitted by WBPH. This discovery not only expands our understanding of the diversity and evolution of *Solemoviridae* viruses but also highlights the potential role of white-backed planthoppers as vectors of novel plant pathogens. Such insights are crucial for developing targeted strategies to manage virus transmission in agricultural ecosystems, ultimately contributing to food security and sustainable crop production.

## Materials and methods

### Sample collection of WBPH

We constructed pooled RNA-Seq libraries for WBPH from nine locations across four provinces in China, respectively. These included Panjin (6A) in Liaoning Province; Sanming (3A) and Zhangzhou (4A and 5A) in Fujian Province; Kunming (16A), Dali Bai Autonomous Prefecture (1A), Honghe Hani and Yi Autonomous Prefecture (2A), and Yuxi (15A) in Yunnan Province; and Beihai (14A) in Guangxi Province, China. WBPH species were identified based on morphological traits. At each site, approximately 30 adult WBPH individuals were collected and pooled for RNA extraction. Sampling sites were chosen to reflect geographic and ecological diversity across major rice production areas in China.

### RNA viromes and small RNA sequencing of WBPH

To characterize the RNA virome and small RNA in WBPH, the RNA of WBPH samples from each location was extracted was extracted using TRIzol reagent (Invitrogen, CA, United States), the extracted RNA was sent to BGI, Wuhan, China, for transcriptomic sequencing (see Supplementary Table S5 for details). Host rRNA was removed and paired-end sequencing (150 bp) of the RNA library was conducted on the DNBSEQ platform for transcriptomic sequencing. The small RNA library was sequenced by the DNBSEQ platform with SE50. The RNA-seq (PRJNA1154385) and small RNA sequencing (SAMN43445664) raw data were downloaded from NCBI.

### Viral sequence and vsiRNA profiles

Following metatranscriptomic sequencing, raw reads were subjected to quality filtering to obtain clean sequences by using fastqc and cutadapt softwares. This process involved removing adaptor sequences, discarding low-quality reads, and eliminating reads containing any ambiguous base calls. Subsequently, clean sequences were *de novo* assembled into longer contiguous sequences (contigs) using MEGAHIT (v1.2.9) with default parameters. These contigs were aligned to the non-redundant protein sequence (nr) database of GenBank^1^ with Diamond BLASTX (v0.9.24.125), employing the *E*-value cutoff of less than 0.001. Only contigs with the best hit of viral protein were retained for further analysis.

Raw small RNA (sRNA) reads were initially processed using Trimmomatic (Bolger et al., 2014) with default settings. Subsequently, the trimmed sRNA reads were mapped to the SoSNV1 reference genome using Bowtie (Langmead et al., 2009), allowing for a maximum of one mismatch. A custom Perl script was utilized to calculate the total counts of sRNAs mapping to both the positive and negative strands of the viral genome, as well as the length distribution of vsiRNAs.

### Virus classification and annotation

Virus-like contigs were clustered using cd-hit-est with a nucleotide identity threshold of 80% to eliminate redundant sequences. The non-redundant contigs were aligned to viral genomes using TBLASTX to evaluate their completeness based on the length ratio (R) between each contig and its best-matched reference. Contigs with R ≥ 0.9 were classified as “complete sequences,” those with 0.8 ≤ R < 0.9 as “near-complete sequences,” and those with R < 0.8 as “fragments.” Subsequently, virus-like contigs were analyzed using NCBI ORFfinder^2^ to predict open reading frames (ORFs), and RPS-BLAST (CD-Search) to identify conserved domains. This information was compared with known viral genome organizations and used as one of the criteria for virus classification. Considering the widespread presence of endogenous viral elements (EVEs) in invertebrates, only complete or near-complete virus-like contigs with typical viral genome organizations were retained for viral species classification and downstream analyses. However, fragments or full-length contigs lacking continuous ORFs and conserved domains were discarded.

Initial virus species classification was based on nucleotide or protein sequence identity between putative viruses and known viruses, as well as the latest species demarcation criteria proposed by the International Committee on Taxonomy of Viruses (ICTV). For viruses lacking established ICTV demarcation criteria, a threshold of 80% nucleotide identity was applied for species-level classification. Contigs sharing ≥80% nucleotide identity with known viruses were assigned accordingly, whereas those falling below this threshold were considered novel viruses. Given that some novel viruses were highly divergent and potentially represented new genera or even families, their taxonomic placement was further evaluated based on their position in RNA-dependent RNA polymerase (RdRp) phylogenetic trees, sequence identity with related members, and genome organization. For multi-segmented viruses, additional segments were determined based on sequence homology, co-occurrence patterns, and relative abundance across sequencing libraries.

### Rapid amplification of cDNA ends

The 5′ rapid amplification of cDNA ends (RACE) was carried out with SMARTer RACE 5′/3′ Kit (Clontech) according to the manufacturer. RNA was treated with SMARTer II A Oligonucleotide prior to reverse transcription. First-strand cDNA synthesis was performed using 5′-CDS primer A and SMART Scribe Reverse Transcriptase. Two rounds of PCR amplification were performed using SeqAmp DNA polymerase (Clonetech), with Universal Primer A Mix and Nested Universal Primer A short (supplied with the kit) as forward primers, and SoSNV1 gene-specific primers (Supplementary Table S6) as reverse primers. The PCR products were subsequently purified, TA-cloned, and sequenced.

To amplify the extreme 3′ end of the cDNA, we utilized the SMARTer RACE 5′/3’ Kit (Clontech). First-strand cDNA synthesis was performed using 3′ RACE CDS primer A, which contains a poly(T) tract, and SMART Scribe Reverse Transcriptase. Two rounds of PCR amplification were performed using SoSNV1 gene-specific primers (Supplementary Table S6) as forward primers and Universal Primer A Mix and Universal Primer A short as reverse primers. The PCR products were subsequently purified, TA-cloned, and sequenced.

### First-strand cDNA synthesis and PCR detection

To investigate the prevalence of SoSNV1 within each library, viral-specific primers were designed using Primer-Blast (NCBI) for the detection of viral genomic RNA (Supplementary Table S6). Total RNA was extracted as previously described, followed by DNase I digestion (Invitrogen) to eliminate genomic DNA contamination. cDNA synthesis was performed using SuperScript^™^ II (Invitrogen) according to the manufacturer’s instructions. PCR products were subsequently verified through sequencing.

### Phylogenetic analysis

Potential ORFs encoding proteins were predicted using NCBI ORFfinder.^3^ For phylogenetic analysis, the conserved RdRp of the novel virus, combined with RdRp protein sequences from reference viruses, were aligned using Maximum likelihood (ML) phylogenetic tree was constructed using MEGA 7.0 (bootstrap = 500) and visualized using Figtree v1.4.0.^4^

## Results

### RNA viromes of WBPH from 9 locations in China

**Figure 1:**
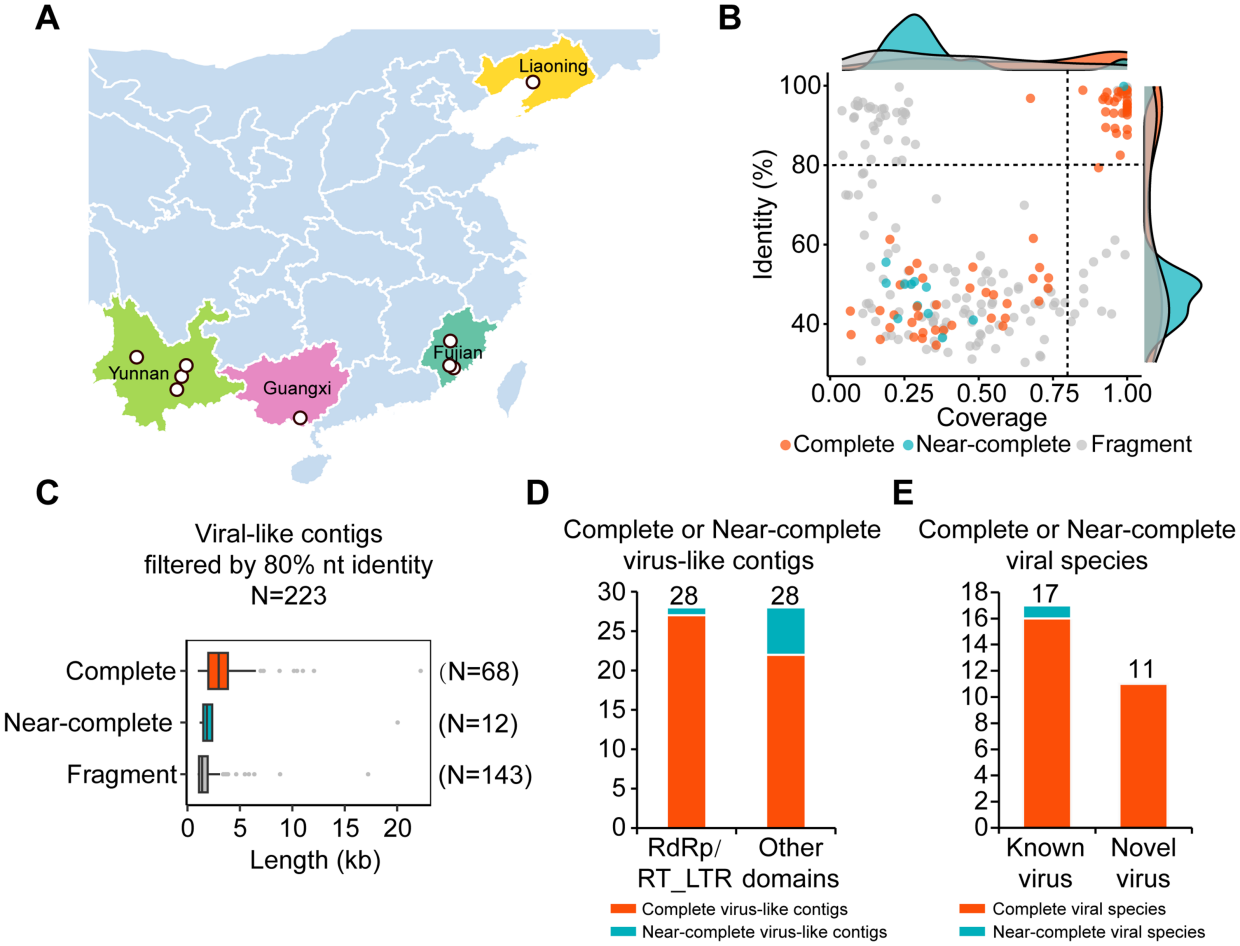
Samples collection and virome analysis. **(A)** Map of sampling locations for white-backed planthoppers. **(B)** Scatter plot showing the identity and coverage of the viral-like contigs **(C)** Histogram of viral-like contigs filtered at 80% nucleotide identity. **(D)** Histogram of complete or near-complete virus-like contigs based on RdRp/RT_LTR conserved domains or other domains. RdRp, RNA-dependent RNA polymerase. RT_LTR, CD1647, reverse transcriptases (RTs) from retrotransposons and retroviruses. Red color indicated the complete viral contigs; green color indicated the near-complete virus-like contigs. **(E)** Histogram of complete or near-complete viral contigs based on known or novel viruses that have RdRp/RT_LTR. Red color indicated the complete viral species; green color indicated the near-complete viral species.

RNA-seq libraries were constructed from WBPH samples collected from nine geographically and ecologically diverse locations across China. These sites were selected to represent four major rice-producing provinces spanning both northern and southern regions, which differ in WBPH abundance and virus prevalence. The sampled locations included Panjin in Liaoning Province; Sanming and Zhangzhou in Fujian Province; Kunming, Dali Bai Autonomous Prefecture, Honghe Hani and Yi Autonomous Prefecture, and Yuxi in Yunnan Province; and Beihai in Guangxi Province, China (Figure 1A). We extracted virus-like contiguous sequences (contigs) longer than 500 nucleotides from these samples and analyzed their coverage and identity (Figure 1B; Supplementary Tables S1, S2). Using a threshold of 80% nucleotide similarity to remove redundant contigs, we identified 223 virus-like contigs. Contigs with a length ratio relative to the most similar viral sequence greater than or equal to 0.9 were defined as complete contigs, those with a ratio greater than or equal to 0.8 but less than 0.9 were defined as near-complete contigs, and those with a ratio less than 0.8 were defined as fragments. According to this standard, we identified 68 complete viral contigs, 12 near-complete viral contigs, and 143 fragments (Figure 1C; Supplementary Table S2). Considering the widespread presence of EVEs in vertebrates, we further analyzed the domains present in the complete and near-complete viral sequences, and discarded fragments and those contigs lacking continuous ORFs and conserved domains. A total of 28 viral sequences (27 complete viral contigs and 1 near-complete viral contig) contained virus-specific RNA-dependent RNA polymerase (RdRp) domains (Zayed et al., 2022) or long terminal repeat reverse transcriptase (RT_LTR) domains (Figure 1D). Another 28 viral sequences (22 complete viral contigs and 6 near-complete viral contigs) contained other domains (Figure 1D). Based on nucleotide similarity and the latest species demarcation proposed by ICTV, contigs with at least 80% nucleotide similarity and a length exceeding 200 nucleotides to reference viruses were classified as known viruses, while contigs with less than 80% similarity or a length less than 200 nucleotides were classified as new viruses identified in this study. Among the 28 complete or near-complete viral contigs containing RdRp or RT_LTR domains, we identified 17 known viruses and 11 novel viruses (Figure 1E).

### Confirmation and abundance of the known and novel viruses

**Figure 2:**
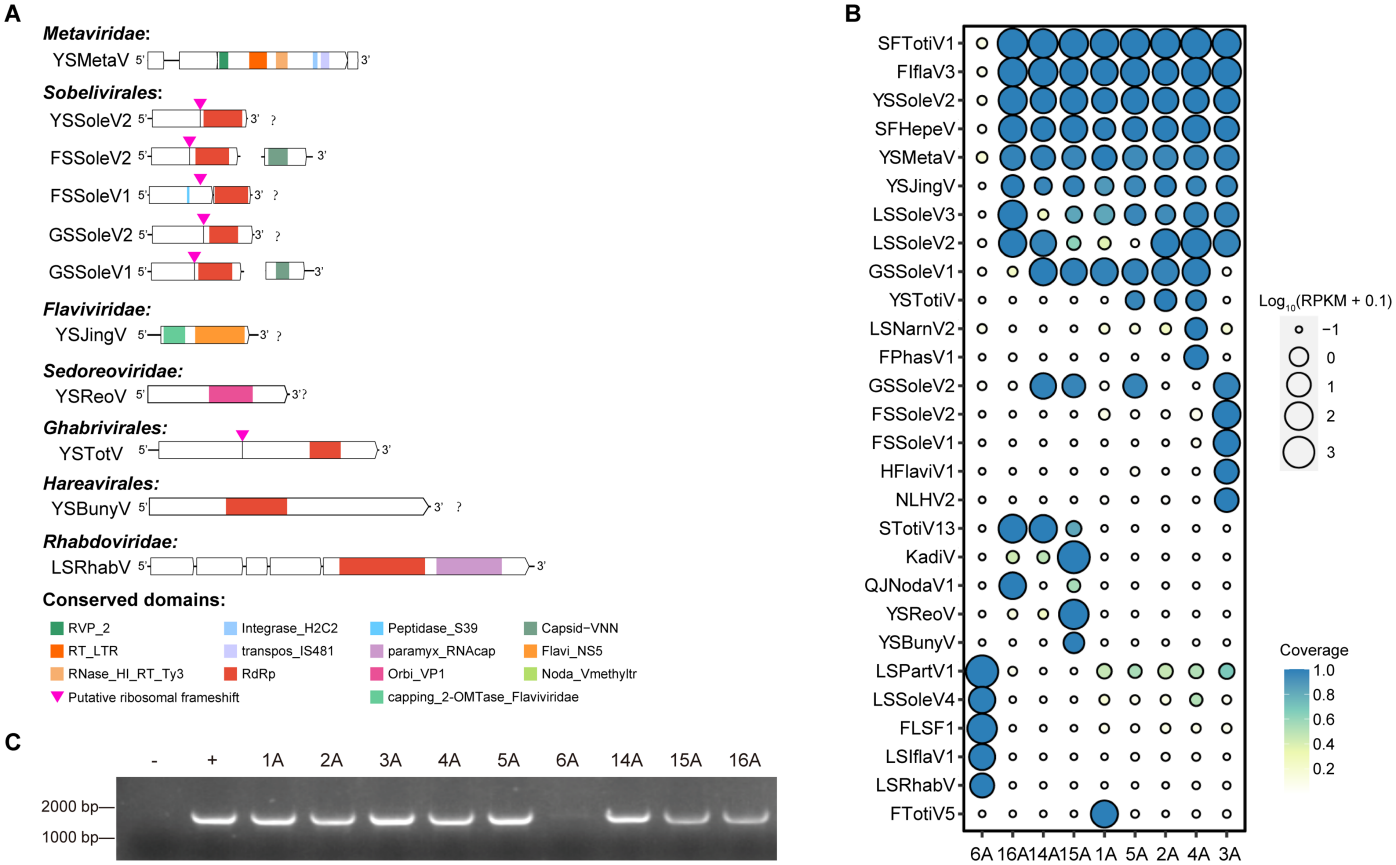
Viral diversity in WBPH samples from different locations. **(A)** Viral genomic structure of 17 novel viruses from 10 distinct viral families. Sequences were listed from 5′ to 3′ orientations. RVP_2: PF08284, Retroviral aspartyl protease. Integrase_H2C2: PF17921, Integrase zinc binding domain. Peptidase_S39: PF02122, Polyprotein processing endopeptidases from RNA viruses. Capsid-VNN: PF11729, nodavirus capsid protein. RT_LTR: CD1647, Reverse transcriptases (RTs) from retrotransposons and retroviruses. Transpos_IS481: NF033577, IS481 family transposase. Paramyx_RNAcap: TIGR04198, mRNA capping enzyme, paramyxovirus family. Flavi_NS5: PF00972, Flavivirus RNA-directed RNA polymerase, fingers and palm domains. RNase_HI_RT_Ty3: CD09274, Ty3/Gypsy family of RNase HI in long-term repeat retroelements. RdRp: PF05183, RNA-dependent RNA polymerase. Orbi_VP1: PF05788, Orbivirus RNA-dependent RNA polymerase (VP1). Noda_Vmethyltr: PF19222, Nodavirus Vmethyltransferase. Capping_2-OMTase_Flaviviridae: CD20761, Cap-0 specific (nucleoside-2’-O-)-methyltransferase of *Flaviviridae*. **(B)** Assessment of RNA-seq read coverage across nine libraries for complete and near-complete viral contigs within the virome. The letter “A” following each location name stands for “Area.” **(C)** Detection of new virus in nine libraries.

Our analysis identified 11 novel viruses spanning 7 distinct viral families, including 7 positive-sense single-stranded RNA viruses (one each from the *Metaviridae* and *Flaviviridae*, 5 from the *Sobelivirales*), 2 negative-sense single-stranded RNA viruses (one each from the *Hareavirales* and *Rhabdoviridae*), and 2 double-stranded RNA viruses (one each from the *Ghabrivirales* and *Sedoreoviridae*) (Figure 2A). We assessed the abundance and coverage of complete and near-complete viral contigs containing RdRp or RT_LTR domains within each library. The results revealed a degree of regional variation in the distribution of these viruses. Notably, the newly identified viruses YSSoleV2, YSMetaV, and YSJingV were particularly abundant and prevalent in WBPH collected from eight locations, excluding Panjin in Liaoning Province. Furthermore, the known viruses SFTotiV1, FIflaV3 and SFHepeV were also relatively abundant and prevalent at these eight locations, excluding Panjin in Liaoning Province. Conversely, LSPartV1, LSSoleV4, FLSF1, LSIflaV1, and LSRhabV were exclusively abundant and prevalent at the Panjin site in Liaoning Province. FTotiV5 was exclusively abundant and prevalent in all Yunnan samples collected at the Dali Bai autonomous prefecture site, while FSSoleV2, FSSoleV1, HFlaviV1, and NLHV2 were exclusively abundant and prevalent in all Fujian samples collected at the Sanming site (Figure 2B; Supplementary Table S3). This suggests that viral distribution can vary even within the same province. Given its high abundance and coverage among *Sobelivirales* viruses, we selected YSSoleV2 for further validation using reverse transcription-polymerase chain reaction (RT-PCR). RT-PCR analysis confirmed the presence of this viral fragment in WBPH collected from eight locations across Guangxi, Fujian, and Yunnan (Figure 2C), corroborating our previous findings. Given that *Solemoviridae* viruses primarily infect plants, this finding suggests that YSSoleV2 could pose a potential threat to crops in southern China.

### Evolutionary relationships of *Sogatella furcifera* novel viruses

**Figure 3:**
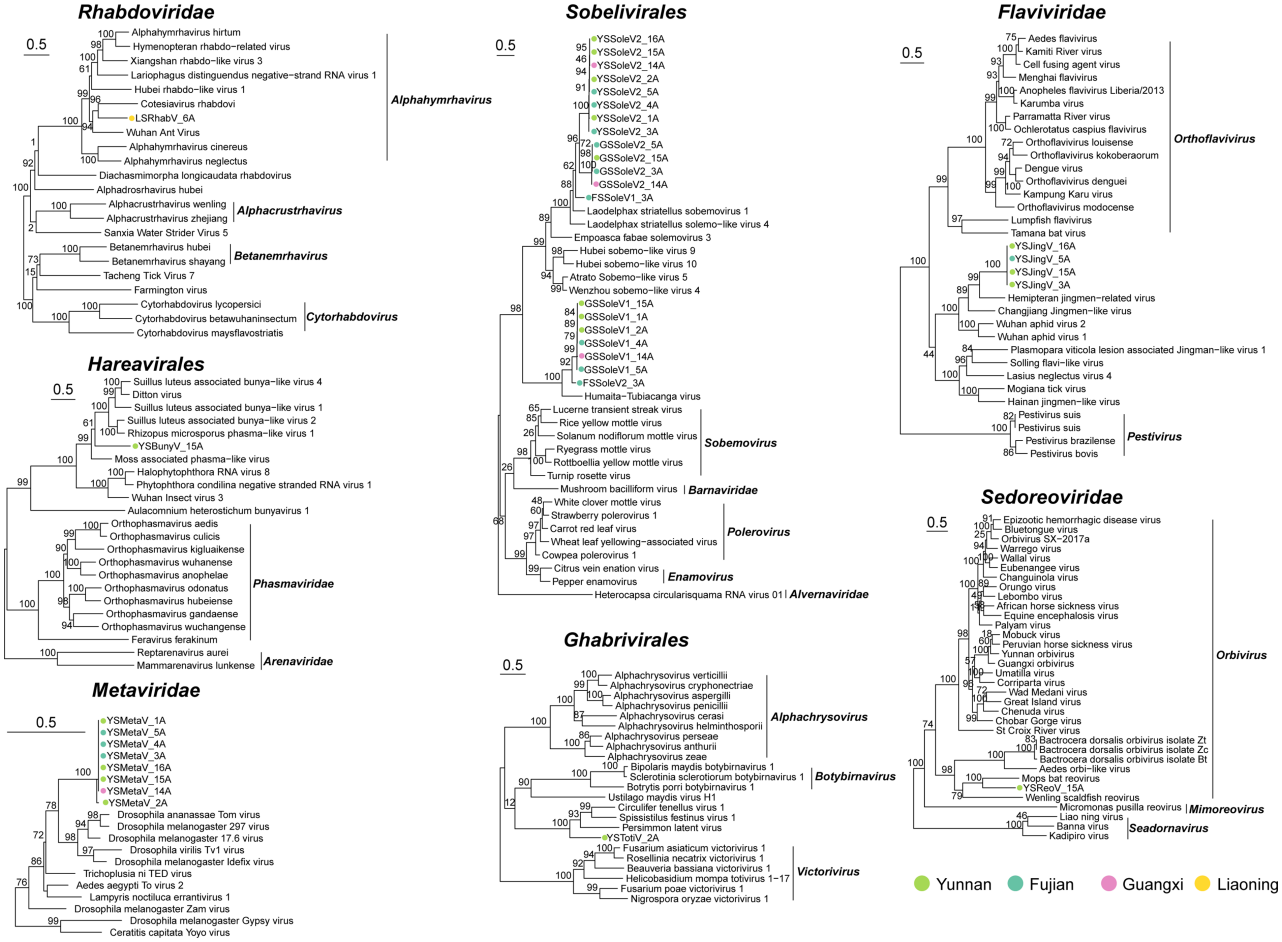
Evolutionary relationship of novel viruses. Maximum-likelihood phylogenetic trees were constructed based on the alignment of the amino acid (aa) sequences of the conserved motifs in the RdRp domain from novel and the selected viruses.

To infer the phylogenetic relationships of the newly identified viruses, we constructed a phylogenetic tree based on the most conserved viral region, RdRp (Figure 3).

Seven putative novel positive-sense RNA viruses were identified and classified into the families *Metaviridae*, *Flaviviridae*, and *Sobelivirales*. The YSMetaV sequence was detected in eight samples (Figure 2B), indicating a close association between *Sogatella furcifera* and this viral group. Phylogenetic analysis revealed that it clusters with *Drosophila* virus (Figure 3). The YSMetaV genome comprises a single segment and includes five conserved domains: RVP_2, RT_LTR, RNase_HI_RT_Ty3, Integrase_H2C2, and transpos_IS481 (Figure 2A). The sequence of YSJingV was also found in eight samples (Figure 2B). Phylogenetic analysis showed that it forms a clade with Hemipteran jingmen-related viruses and contains the capping_2-OMTase domain, typical of *Flaviviridae* (Figures 2A, 3). Studies have shown that positive-strand RNA viruses, including jingmen viruses, originated from an ancestor with an E glycoprotein and cap-dependent translation, thereby necessitating the presence of MTase. The presence of the E glycoprotein and MTase suggests that the virus relies on a cap-dependent translation mechanism. Viruses of this type can infect a wide range of hosts and are frequently associated with vector-borne transmission between vertebrates and invertebrates, such as mosquitoes (Mifsud et al., 2024). The discovery of YSJingV in *Sogatella furcifera* suggested its potential for cross-species transmission. Among the viral sequences assigned to *Sobelivirales*, a cluster of three highly similar acidic sequences sharing the same genome structure grouped with *Laodelphax striatellus* sobemovirus 1 and *Laodelphax striatellus* solemo-like virus 4 (Figure 3). The genomes of YSSoleV2, GSSoleV2, and FSSoleV2 consist of a single segment encoding one ORF, which harbors the conserved RdRp domain and a putative ribosomal frameshift (Figure 2A). Two additional novel viral sequences belonging to *Sobelivirales* clustered with a previously described RNA virus (Humaita-Tubiacanga virus) identified in mosquitoes (Shi et al., 2019) (Figure 3). The genomes of GSSoleV1 and FSSoleV2 consist of two segments encoding two ORFs, which include the conserved domains RdRp and Capsid-VNN, along with a putative ribosomal frameshift (Figure 2A).

The two putative novel negative-sense RNA viral sequences were assigned to the orders *Hareavirales* and *Rhabdoviridae*. Among the viral sequences classified under *Rhabdoviridae*, LsRhabV_6A, detected exclusively in *Sogatella furcifera* samples from Panjin, Liaoning Province (Figure 2B), clustered with *Alphaymrhavirus*. Phylogenetic analysis indicated that it forms a clade with Cotesiavirus rhabdovi and Wuhan Ant virus (Figure 3). The LsRhabV genome comprises a single segment encoding the conserved domains RdRp and paramyx_RNAcap (Figure 2A). In contrast, YSBunyV, classified under *Hareavirales* and detected only in *Sogatella furcifera* samples from Yuxi, Yunnan Province (Figure 2B), closely clustered with Suillus luteus-associated bunya-like virus and Rhizopus microspores phama-like virus (Figure 3), suggesting its potential classification as a fungal virus. The YSBunyV genome encodes the conserved RdRp domain (Figure 2A).

We identified two double-stranded RNA viral sequences, one belonging to *Ghabrivirales* and the other to *Orbivirus* (Figure 3). YSTotiV clustered with two insect viruses, *Circulifer tenellus* virus 1 and *Spissistilus festinus* virus 1. The YSTotiV genome encodes the conserved RdRp domain and a putative ribosomal frameshift (Figure 2A). One *Orbivirus* (YSReoV) clustered with Mops bat virus (Figure 3). *Orbiviruses* are arthropod-borne viruses with a 10-segmented double-stranded RNA genome, transmitted by vectors such as mosquitoes, midges, and ticks (Matthijnssens et al., 2022). YSReoV was identified exclusively in samples collected from Panjin, Liaoning Province (Figure 2B), although only one genome segment was detected, encoding the conserved domains RdRp and paramyx_RNAcap (Figure 2A).

### Sequence characteristics and domain prediction of *Sogatella furcifera* solemo-like virus 1

**Figure 4:**
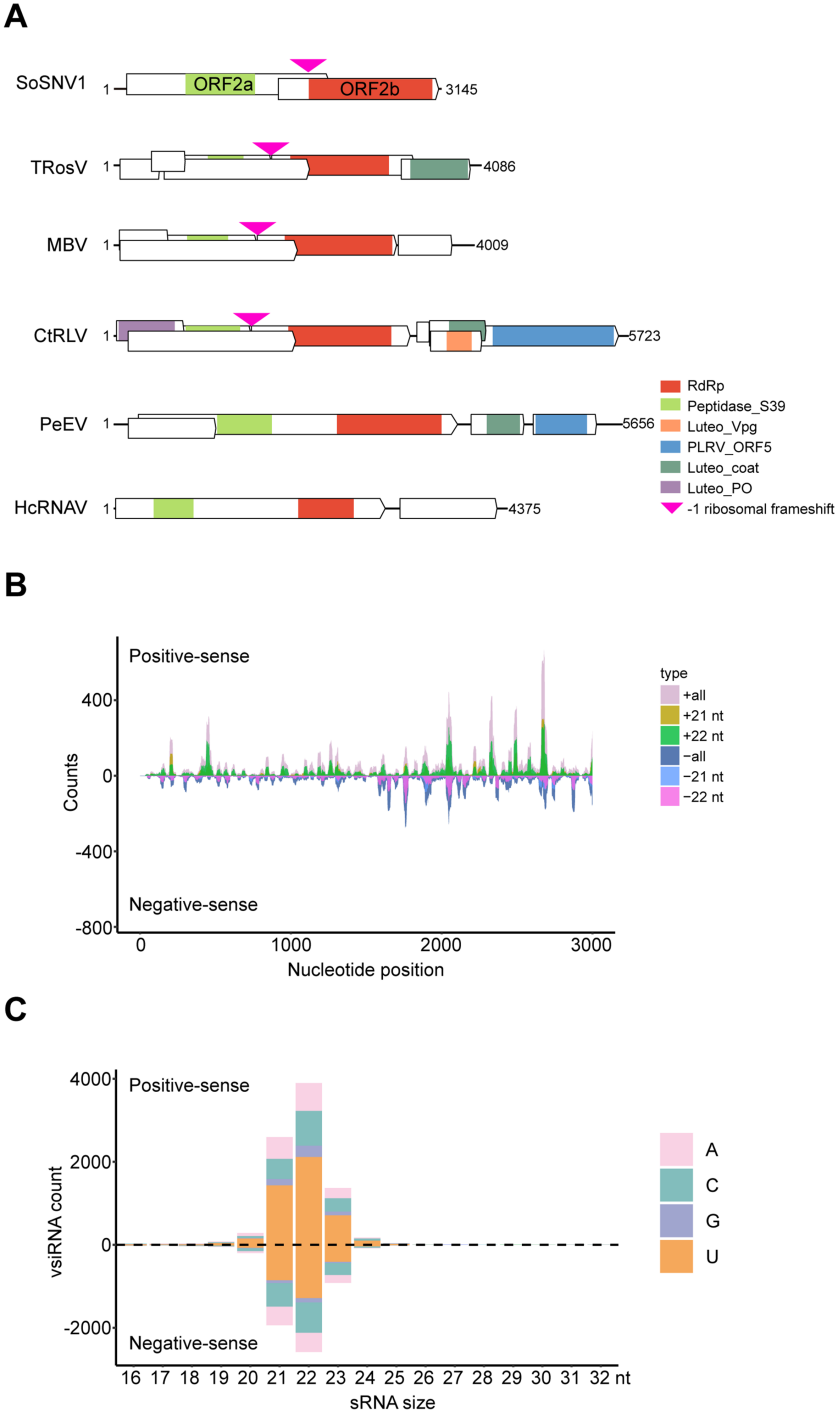
Genomic structure and vsiRNA profile of SoSNV1. **(A)** Viral genomic structure of SoSNV1 and its comparative analysis with closely related viruses. TRoSV, turnip rosette virus, NC_004553.3. MBV, mushroom bacilliform virus, NC_001633.1. CtRLV, carrot red leaf virus, NC_006265.1. PeEV, pepper enamovirus, NC_037052.1. HcRNAV, heterocapsa circularisquama RNA virus, NC_007518.1. RdRp: PF05183, RNA-dependent RNA polymerase. Peptidase_S39: PF02122, Polyprotein processing endopeptidases from RNA viruses. Luteo_Vpg: PF01659, *Luteovirus* putative VPg genome linked protein. PLRV_ORF5: PF01690, potato leaf roll virus readthrough protein. Luteo_coat: PF00894, *Luteovirus* coat protein. Luteo_P0: PF04662, *Luteovirus* P0 protein. **(B)** Distribution of vsiRNAs corresponding to the viral genome of SoSNV1. **(C)** Distribution of vsiRNAs from the positive strand and negative strand in length. nt, nucleotide.

The *Solemoviridae* is another important family of plant linear (+) ssRNA viruses that are divided into four genera (*Enamovirus*, *Polemovirus*, *Polerovirus*, and *Sobemovirus*).^5^ Given the potential of *Solemoviridae* to infect Gramineae plants and the widespread presence of YSSoleV2 in southern China, we focused our analysis on YSSoleV2. Through sequencing and assembly of WBPH samples from Guangxi, Fujian, and Yunnan, we obtained a nearly full-length sequence of YSSoleV2 (Supplementary Table S3). After obtaining the terminal sequences using the RACE technique, the full-length sequence was determined to be 3,145 nt and was subsequently renamed SoSNV1 (Supplementary Table S4) (GenBank: PQ283853). Genome analysis suggests that SoSNV1 encodes two proteins: ORF2a and ORF2b (Figure 4A). ORF2a is predicted to encode a 598-amino acid protein, while ORF2b is predicted to encode a 987-amino acid protein produced through a −1 ribosomal frameshift. ORF2a and ORF2b may be cleaved into three segments each. The first segment of ORF2a is predicted to be involved in membrane anchoring. The remaining segments have unknown functions but may be involved in other viral processes. The C-terminal of ORF2b is predicted to function as an RNA-dependent RNA polymerase (Figure 4A). To further investigate the structural divergence of SoSNV1 within the order *Sobelivirales*, we compared its genome organization with that of five representative members: turnip rosette virus (TRosV) (NC_004553.3), mushroom bacilliform virus (MBV) (NC_001633.1), carrot red leaf virus (CtRLV) (NC_006265.1), pepper enamovirus (PeEV) (NC_037052.1), and heterocapsa circularisquama RNA virus (HcRNAV) (NC_007518.1) (Figure 4A). All of these viruses were phylogenetically related to SoSNV1 based on RdRp sequences (Figure 3). SoSNV1 possesses a compact genome comprising only two ORFs. In contrast, the other viruses (TRosV, MBV, CtRLV, PeEV, HcRNAV) exhibit larger genomes and more complex organizations. Notably, SoSNV1 lacks any identifiable viral genome-linked protein (VPg), coat protein, or movement protein coding regions, which are present in at least some of the comparator viruses. These structural differences, along with phylogenetic distinctness, suggest that SoSNV1 may as a candidate for a new genus within *Sobelivirales*.

Viral dsRNA replication intermediates and double-stranded structures derived from the viral genome can induce a host RNA interference (RNAi) response, leading to the generation of viral small interfering RNAs (vsiRNAs) and subsequent silencing of the viral genome. To characterize the vsiRNAs associated with SoSNV1 infection, small RNA libraries were constructed from WBPH. Analysis of these libraries revealed nearly equal proportions of vsiRNAs derived from both the positive and negative strands of the viral genome, with the exception of several asymmetric hotspots (Figure 4B) that may play critical roles in host-virus interactions. Furthermore, the vsiRNAs exhibited a typical size distribution and polarity, with a predominant length of 21 and 22 nucleotides and a strong 5′ terminal preference for uracil (U), followed by cytosine (C), adenine (A), and guanine (G) (Figure 4C). These characteristic vsiRNA profiles indicate active viral replication within the WBPH host and suggest that the host’s antiviral RNAi pathway is likely responding to the viral infection.

## Discussion

The discovery of viral genomes through large-scale metagenomic sequencing and transcriptome analysis has facilitated rapid virus identification and expanded our understanding of viral diversity (Backstrom et al., 2019; Schulz et al., 2022; Schulz et al., 2020; Shi et al., 2018; Song et al., 2021). Metagenomic studies of insects have revealed numerous insect-specific viruses (Liu et al., 2015; Liu et al., 2011; Nouri et al., 2018; Shi et al., 2016), some of which belong to the same families as plant viruses, raising concerns about their potential to evolve into plant pathogens (Somera et al., 2021). The virome of *Sogatella furcifera* remains a critical yet underexplored component of its ecology and role as an agricultural pest. Previous studies have provided valuable insights into the viral diversity associated with WBPH, they have primarily focused on specific virus families or relied on publicly available sequencing datasets (Wu et al., 2018a; Wu et al., 2018b; Yuan et al., 2024; Zhang et al., 2018). These approaches have been instrumental in identifying novel viruses, yet the full range of viruses present in natural WBPH populations remains insufficiently explored. In this study, we employed a meta-transcriptomic approach on field-collected WBPH samples from nine geographically diverse locations across China, allowing for a direct, unbiased assessment of the RNA virome in natural WBPH populations. Our dataset enabled the identification of 223 viral contigs, including 11 novel viruses across seven viral families, substantially expanding the known WBPH virome. Notably, our study identified single-stranded positive-sense RNA, negative-sense RNA, and double-stranded RNA viruses, underscoring the diverse viral community harbored by WBPH.

Recent research efforts have leveraged publicly available sequencing datasets to explore the virome of WBPH, providing a powerful means of uncovering viral sequences across a wide range of sequencing projects (Yuan et al., 2024). However, our study utilized field-collected WBPH samples, ensuring a more systematic and regionally representative dataset for virome analysis. This approach allowed us to identify viruses that might not be well-represented in public databases, particularly those with low abundance, regional specificity, or associations with particular ecological conditions. One key example is our identification of SoSNV1, a novel *Solemoviridae* virus, in WBPH populations across Guangxi, Fujian, and Yunnan. While *Solemoviridae* viruses are typically plant-infecting (Dolja et al., 2020; Guo et al., 2022; Sarra and Peters, 2003; Traore et al., 2009), their presence in WBPH suggests a potential vector role for this pest in plant virus transmission. Importantly, prior research has also identified three Solemo-like viruses in WBPH using public database analyses, providing additional evidence that these viruses are consistently associated with this species.

*Solemoviridae* members are categorized into four genera and an unclassified portion (Walker et al., 2021). All members possess RdRp and −1 ribosomal frameshift sites, although their structural domains vary (Somera et al., 2021; Somera et al., 2015). Structural domain prediction shows that, in comparison to other members of the same family, SoSNV1 lacks VPg, ORF1, and ORF3 proteins. Previous studies have shown that *Sobemovirus* ORF1 functions as an RNA silencing suppressor and ORF3 as a coat protein (Somera et al., 2021; Somera et al., 2015), both of which are essential for the infection process (Csorba et al., 2015). The absence of these essential proteins may be attributed to incomplete evolutionary development, potentially preventing the virus from infecting plants and explaining its limited prevalence in the wild. Therefore, we believe that regular monitoring and forecasting of common vectors is crucial, as these practices can provide early warnings of epidemics and facilitate timely control measures.

The evolutionary relationships of the newly identified viruses suggest complex interactions between WBPH, plant hosts, and other insect-associated viruses. Our study uncovered viruses such as YSBunyV and LsRhabV, which group with known negative-sense RNA viruses found in both insects and fungi. This raises intriguing questions about the possible horizontal gene transfer and host-switching events that may have shaped the evolution of WBPH-associated viruses (Gilbert and Cordaux, 2017). Given that many plant viruses rely on insect vectors for transmission, understanding these evolutionary relationships could provide insights into how WBPH-associated viruses adapt to different host environments. Additionally, the presence of insect-associated double-stranded RNA viruses (YSTotiV and YSReoV) in our dataset suggests that WBPH may harbor a more ecologically diverse virome than previously recognized. The detection of these viruses, which cluster with known insect and fungal dsRNA viruses, supports the hypothesis that arthropods play an important role in the persistence and dissemination of diverse viral taxa (Marklewitz and Junglen, 2019; Perveen et al., 2023). In addition, environmental factors such as agrochemical input, host plant variety, and cropping intensity may influence the virome composition of WBPH. Future studies that integrate ecological metadata could further refine our understanding of virus-vector-environment interactions.

As an important rice pest, WBPH has well-established roles in transmitting plant viruses such as Southern rice black-streaked dwarf virus (SRBSDV) (Wang et al., 2022; Zhou et al., 2013; Zhou et al., 2008). Our discovery of multiple *Solemoviridae*-like viruses in WBPH suggests that additional, previously uncharacterized viruses may also be involved in plant-virus transmission. Although several of the detected viruses are phylogenetically related to taxa that include plant- or fungus-associated members, we did not identify any viruses with known pathogenicity to plants or evidence of cross-kingdom transmission. At this stage, these viruses should be considered insect-associated. Further studies-such as host range tests, tissue localization, and transmission assays—are needed to explore their biological functions, tissue tropism, and ecological impact. Given the potential economic impact of these viruses (Somera et al., 2021), further research is required to assess whether they contribute to plant disease outbreaks and how they may affect crop health. Furthermore, our small RNA sequencing analysis of SoSNV1 revealed a characteristic 21–22 nt viral small interfering RNA (vsiRNA) profile, with a strong 5′ uracil (U) preference, indicative of an active RNAi immune response in WBPH (Baulcombe, 2022; Bonning and Saleh, 2021). This suggests that the host-virus interactions in WBPH may influence viral persistence, transmission efficiency, and co-evolutionary dynamics. Understanding the immune response of WBPH to its associated viruses is crucial for deciphering the mechanisms of viral maintenance and spread in natural populations.

Our study highlights the complex virome of WBPH and its potential implications for virus-vector interactions, crop health, and cross-species transmission. Future research should focus on experimental infections, viral metagenomics, and functional genomics to determine the biological significance of these viruses, their transmission mechanisms, and their impact on plant health. Additionally, studies investigating host-virus co-evolution and spillover events will be essential for understanding viral adaptation in Hemiptera species. Given the agricultural importance of WBPH, a deeper investigation into its virome could provide valuable insights into pest management strategies, particularly in controlling vector-borne plant diseases.

By integrating field-collected WBPH samples with meta-transcriptomic analysis, our study provides a comprehensive perspective on the viral diversity within this economically important insect vector. The identification of novel viruses across diverse families, including potential plant-infecting and cross-species transmission candidates, underscores the ecological and agricultural significance of the WBPH virome. Our findings complement previous research based on public database analyses, demonstrating the added value of direct field sampling in revealing the natural diversity of insect-associated viruses. These results lay the foundation for future studies into host-virus interactions, viral evolution, and the role of insect vectors in virus transmission, ultimately contributing to better pest and disease management strategies in agricultural ecosystems.

## Data Availability

The datasets presented in this study can be found in online repositories. The names of the repository/repositories and accession number(s) can be found in the article/Supplementary material.

## Footnotes

^1^ http://www.ncbi.nlm.nih.gov/

^2^https://www.ncbi.nlm.nih.gov/orffinder, accessed on 22 February 2024.

^3^https://www.ncbi.nlm.nih.gov/orffinder, accessed on 22 February 2024.

^4^ http://tree.bio.ed.ac.uk/software/figtree/

^5^https://talk.ictvonline.org/taxonomy/, accessed on 20 August 2021.
